# Репликативные и биохимические аспекты старения у женщин с преждевременной недостаточностью яичников

**DOI:** 10.14341/probl13253

**Published:** 2023-05-12

**Authors:** Р. К. Михеев, Е. Н. Андреева, О. Р. Григорян, Е. В. Шереметьева, Ю. С. Абсатарова, Е. В. Логинова

**Affiliations:** Национальный медицинский исследовательский центр эндокринологии; Национальный медицинский исследовательский центр эндокринологии; Московский государственный медико-стоматологический университет им. А.И. Евдокимова; Национальный медицинский исследовательский центр эндокринологии; Национальный медицинский исследовательский центр эндокринологии; Национальный медицинский исследовательский центр эндокринологии; Российский университет дружбы народов

**Keywords:** преждевременная недостаточность яичников, теломеры, биохимический анализ, ФСГ, сахарный диабет, предиабет

## Abstract

**ОБОСНОВАНИЕ:**

ОБОСНОВАНИЕ. Одной из наиболее опасных патологий с точки зрения демографического урона, ухудшения качества жизни и снижения шансов на здоровое долголетие у женщин является преждевременная недостаточность яичников (ПНЯ). Помимо манифестации до 40 лет, данному симптомокомплексу присуще наличие вторичной аменореи, тотального эстрогенного дефицита и гипергонадотропного гипогонадизма. Существуют небезосновательные опасения о влиянии ПНЯ на ожидаемую продолжительность жизни за счет пагубного действия эстрогенного дефицита на длину теломер. Многообещающими мерами по сохранению качества и ожидаемой продолжительности жизни у пациенток с ПНЯ являются определение длины теломер и инициация гормональной заместительной терапии.

**ЦЕЛЬ:**

ЦЕЛЬ. Изучить особенности репликативного клеточного старения (длины теломер) и биохимических показателей у женщин с ПНЯ.

**МАТЕРИАЛЫ И МЕТОДЫ:**

МАТЕРИАЛЫ И МЕТОДЫ. Исследование проведено на базе ФГБУ «НМИЦ эндокринологии» Минздрава России совместно с МНОЦ МГУ им. М.В.Ломоносова в период с 10.01.2021 г. по 01.08.2022 г.

В одномоментном сравнительном исследовании приняли участие 33 женщины с неятрогенным гипергонадотропным гипогонадизмом в исходе ПНЯ и 24 здоровые женщины репродуктивного возраста (18–49 лет). Пациенткам проведен лабораторный генетический (длина теломер лейкоцитов), биохимический анализы крови. Экстракция ДНК — набором Qiagen DNA blood mini kit (Германия). Оценка длины теломер лейкоцитов — методом полимеразной цепной реакции (ПЦР) в реальном времени (алгоритм Flow-fish).

**РЕЗУЛЬТАТЫ:**

РЕЗУЛЬТАТЫ. Женщины с ПНЯ вследствие эстрогенного дефицита имеют незначительно более низкую среднюю длину теломер (10,0 [7,9–10,7] кБ, чем здоровые пациентки репродуктивного возраста (10,8 [10,0–13,1] кБ, р<0,001). Пациентки с ПНЯ вследствие эстрогенного дефицита имеют склонность к развитию нарушений углеводного обмена (предиабету) (р<0,043) и повышению уровня фолликулостимулирующего гормона (ФСГ) (р<0,001). Уровень ФСГ умеренно отрицательно (ρ=0,434) коррелирует с длиной теломер лейкоцитов у женщин (р<0,001).

**ЗАКЛЮЧЕНИЕ:**

ЗАКЛЮЧЕНИЕ. На фоне приема заместительной гормональной терапии у пациенток с ПНЯ в отличие от здоровых женщин репродуктивного возраста отмечаются снижение длины теломер и повышение риска развития нарушений углеводного обмена.

## Abstract (en)

**BACKGROUND:**

BACKGROUND: One of the most dangerous reproductive pathologies is primary ovarian insufficiency (POI). Except manifestation in the age <40 years old it leads to demographical losses, decrease of chances for healthy aging. POI can be characterized as summary of secondary amenorrhea, total estrogenic deficiency and hypergonadotropic hypogonadism. Hence, POI has probably harmful effect on telomere length. Telomere length determining and sex steroid replacement therapy may be promising and effective to prevent decrease of life quality/ longevity among females with POI.

**AIM:**

AIM: To evaluate features of replicative (telomere length) and biochemical (metabolic syndrome) markers among females with primary ovarian insufficiency.

**MATERIALS AND METHODS:**

MATERIALS AND METHODS. Research has been provided in collaboration between Endocrinology Research Centre of the Russian Ministry of Health and Lomonosov Moscow State University Medical Research and Educational Centre in the period since 10.01.2021 until 01.08.2022. Females with non-iatrogenic hypergonadotropic hypogonadism caused by primary ovarian insufficiency (n=33); healthy females of reproductive age (18–49 y.o.; n=24). Patients have undergone laboratory genetic (leucocyte telomere length), biochemical analyses. DNA extraction — with Qiagen DNA blood mini kit (Germany).

Leukocyte telomere length — with real-time polymerase chain reaction PCR (Flow-fish). Soft program IBM SPSS Statistics (version 26,0 for Windows).

**RESULTS:**

RESULTS. Females with POI due to estrogenic deficiency have slightly shorter mean telomere length (10,0 [7,9–10,7] kB, than healthy females of reproductive age (10,8 [10,0–13,1] кБ, р<0,001). Females with POI due to estrogenic deficiency have higher chances for development of carbohydrate metabolism disturbances (prediabetes) (р<0,043), increasement of FSH level (р<0,001). FSH level correlates moderately and negatively (ρ=0,434) with leukocyte telomere length (р<0,001).

**CONCLUSIONS:**

CONCLUSIONS. Female with POI and receiving sex steroid replacement therapy have decrease of telomere length and increase of chances for carbohydrate metabolism disturbances in opposite to healthy reproductive females.

## ОБОСНОВАНИЕ

Одной из наиболее актуальных и наименее изученных проблем современной репродуктивной эндокринологии является лечебное диагностическое сопровождение пациенток с преждевременной недостаточностью яичников. Отличительными особенностями данного симптомокомплекса являются вторичная аменорея, симптомы дефицита эстрогенов (вазомоторные проявления, генитоуринарный синдром) и гипергонадотропный гипогонадизм (повышение уровней фолликулостимулирующего (ФСГ) и лютеинизирующего (ЛГ) гормонов) с манифестацией до 40 лет у ранее репродуктивно-сохранных женщин [1]. Лабораторная верификация данной нозологии основана на получении минимум дважды результатов уровня ФСГ >25 МЕ/л с интервалом не менее 4–6 нед у пациенток с аменореей на протяжении не менее 4 мес [2]. Несмотря на 80-летнюю историю изучения данного синдрома, начало которой было положено Albright F. и соавт. (1942) [3], этиология и патогенез развития преждевременной недостаточности (ПНЯ) остаются до сих пор не изученными. Существует множество теорий, объясняющих преждевременное истощение фолликулярного аппарата яичников, ни одна из которых не признана исчерпывающей; примером тому являются генетическая (премутация гена FMR1 [1], мутации генов BMP15, LHR, FSHR, GDF9, STAG3 [2], низкий уровень экспрессии Tet1 [4]), аутоиммунная (антитела к 21-гидроксилазе, тиреопероксидазе, рецепторам тиреотропного гормона) [5] и инфекционная (эпидемический паротит, COVID-19) [6][7] теории. Установлено, что сочетание генетической предрасположенности с экзогенными факторами (инфекционные агенты, стрессы, ионизирующее излучение) способно вызывать повреждение гонад с последующим замещением фолликулов соединительной тканью. Процесс апоптоза фолликулов лабораторно проявляется в виде снижения уровня антимюллерова гормона (АМГ) и эстрогенов с исходом в бесплодие и тотальный эстрогенный дефицит соответственно; последнее состояние даже при ранней (до 40 лет) манифестации задолго до вступления в пожилой возраст способствует развитию заболеваний, ассоциированных со старением (атеросклероз, артериальная гипертония, дислипидемия, инсулинорезистентность, сердечно-сосудистые, неврологические, костно-мышечные заболевания) [8]. Общепринятой терапевтической мерой при развитии ПНЯ является заместительная терапия половыми стероидами (в частности, эстрадиолом) [8], которая путем воздействия на эстрогеновые рецепторы ERα и ERβ [9] блокирует дегенеративные изменения старческого генеза в различных органах и системах. Однако в свете современных представлений о так называемой теломеразной теории старения большое внимание уделяется гипотезе о протективном влиянии эстрогенов на состояние «биологических часов» клетки — теломер.

Теломеры (от др.-греч. τέλος — конец + μέρος — часть) служат концевыми структурами ДНК, состоящими из тандемных повторов нуклеотидных последовательностей TTAGGG на 3’-конце в сочетании с ассоциированными белками-шелтеринами, в частности TPP1 [10]. В течение жизненного цикла клетки теломеры поддерживают стабильность ДНК за счет ревертазной активности теломеразы — фермента рибонуклеиновой природы, включающего в себя теломеразную обратную транскриптазу (hTERT), и некодирующей РНК (hTR) [11]. По данным L. Gaydosh и соавт. (2020), эстрогены показали статистически значимое протективное влияние на теломеры как in vitro, так и in vivo [12], что подтверждается достижением более выраженной длины теломер к концу пубертата у женщин по сравнению с мужчинами (исходный показатель при рождении: 7,01±0,03 kb против 6,87±0,04 kB, наблюдение (мужчины): 6,79±0,03 против 6,65±0,03, Р=0,005) [13].

Авторы настоящего исследования выдвигают гипотезу, в рамках которой утверждается способность эстрогенов в составе заместительной терапии обеспечивать длину теломер либо превышающую, либо сопоставимую с аналогичным показателем у здоровых женщин репродуктивного возраста (15–49 лет).

## ЦЕЛЬ ИССЛЕДОВАНИЯ

Изучить особенности маркера репликативного клеточного старения (длина теломер) и биохимических показателей у женщин с ПНЯ на фоне заместительной терапии половыми стероидами.

## МАТЕРИАЛЫ И МЕТОДЫ

Место и время проведения исследования

Исследование проведено на базе ФГБУ «НМИЦ эндокринологии» Минздрава России совместно с МНОЦ МГУ им. М.В.Ломоносова в период с 10.01.2021 г. по 01.08.2022 г.

Изучаемые популяции

В одномоментном сравнительном исследовании всего приняли участие 57 женщин, из них:

1.33 женщины (<40 лет) с ПНЯ на гормональной заместительной терапии (ГЗТ);

2.24 здоровые женщины репродуктивного возраста (15–49 лет), не получающие ГЗТ.

Изучаемые популяции пациентов

I.Пациентки с установленным диагнозом «Преждевременная недостаточность яичников», получающие заместительную терапию половыми стероидами >5 лет (основная группа).

Критерии включения:

пациентки женского пола паспортного возраста <40 лет, находящиеся в состоянии аменореи длительностью не менее 5 лет; подтвержденный медицинской документацией факт получения ГЗТ в дозе эстрогенового компонента 2 мг в течение не менее 5 лет. Все пациентки подписывали информированное согласие на проведение обследования и консультирование.

Критерии исключения.

1.Наличие ятрогенной менопаузы в анамнезе:

1.1.после перенесенных хирургических вмешательств;

1.2.после перенесенной химиотерапии;

1.3.после перенесенной лучевой терапии;

1.4.после комбинированного лечения из вышеперечисленных выше методов.

2.Наличие сопутствующей патологии в анамнезе:

2.1.генетическая патология репродуктивной системы (синдром Тернера, кариотип 45, XO/XY);

2.2.эстроген-зависимые заболевания и патологические состояния (гиперпластические процессы эндометрия, миома матки, все формы эндометриоза);

2.3.нарушения функции щитовидной железы;

2.4.наличие официально задокументированных психических расстройств;

2.5.наличие официально задокументированных злокачественных новообразований;

2.6.нарушения углеводного обмена (нарушенная толерантность к глюкозе, нарушенная гликемия венозной плазмы натощак, сахарный диабет 1 и 2 типов);

2.7.сердечно-сосудистые заболевания (ишемическая болезнь сердца, острое нарушение мозгового кровообращения, тромбоэмболия легочной артерии).

3.Другие физиологические состояния репродуктивной системы:

3.1.беременность;

3.2.период грудного вскармливания.

Способ формирования выборки — произвольный.

II.Здоровые женщины репродуктивного возраста без заболеваний репродуктивной системы, не получающие МГТ (контрольная группа).

Критерии включения:

пациентки женского пола паспортного возраста 20–49 лет с сохраненным менструальным циклом; уровень ФСГ в фолликулярную фазу — в пределах 2,0–11,6 МЕ/л, в лютеиновую фазу — в пределах 1,4–9,6 МЕ/л. Все пациентки подписывали информированное согласие на проведение обследования и консультирование.

Критерии исключения.

1.Наличие физиологической менопаузы в анамнезе.

2.Наличие ятрогенной менопаузы в анамнезе:

2.1.после перенесенных хирургических вмешательств;

2.2.после перенесенной химиотерапии;

2.3.после перенесенной лучевой терапии;

2.4.После комбинированного лечения из вышеперечисленных выше методов.

3.Наличие сопутствующей патологии в анамнезе:

3.1.генетическая патология репродуктивной системы (синдром Тернера, кариотип 45, XO/XY);

3.2.наличие аутоиммунной менопаузы в анамнезе (в исходе первичной недостаточности яичников);

3.3.эстроген-зависимые заболевания и патологические состояния (гиперплазия эндометрия, миома матки, все формы эндометриоза);

3.4.нарушения функции щитовидной железы;

3.5.наличие официально задокументированных психических расстройств;

3.6.наличие официально задокументированных злокачественных новообразований;

3.7.нарушения углеводного обмена (нарушенная толерантность к глюкозе, нарушенная гликемия венозной плазмы натощак, сахарный диабет 1 и 2 типов);

3.8.сердечно-сосудистые заболевания ишемическая болезнь сердца, острое нарушение мозгового кровообращения, тромбоэмболия легочной артерии));

4.Другие физиологические состояния репродуктивной системы:

4.1.беременность;

4.2.период грудного вскармливания.

Способ формирования выборки — произвольный.

Описание вмешательства

Пациенткам проведены лабораторный генетический (длина теломер лейкоцитов), биохимический анализы.

Экстракция ДНК проведена набором Qiagen DNA blood mini kit (Германия).

Оценка длины теломер лейкоцитов — методом полимеразной цепной реакции (ПЦР) в реальном времени (алгоритм Flow-fish).

Дизайн исследования

Обсервационное одномоментное сравнительное активное ретроспективное.

Статистический анализ

Статистическая обработка данных выполнена с помощью программы IBM SPSS Statistics (version 26,0 for Windows).

Категориальные переменные представлены в виде абсолютных и относительных частот. Количественные показатели оценивались на предмет соответствия нормальному распределению с помощью критерия Шапиро–Уилка. В случае отсутствия нормального распределения количественные данные описывались с помощью медианы (Me) и нижнего и верхнего квартилей (Q1–Q3). При нормальном распределении — средними значениями и стандартным отклонением. Сравнение двух групп по количественному показателю, распределение которого отличалось от нормального, выполнялось с помощью U-критерия Манна–Уитни. При нормальном распределении использовался t-критерий Стьюдента. Анализ многопольных таблиц сопряженности выполнялся с помощью точного критерия Фишера. Корреляция между переменными была проверена с помощью корреляционного анализа по методу ρ Спирмена. Прогностическая модель, характеризующая зависимость количественной переменной от факторов, разрабатывалась с помощью метода линейной регрессии.

Этическая экспертиза

Протокол исследования был одобрен этическим комитетом ФГБУ «НМИЦ эндокринологии» Минздрава России (№11 от 22.07.2021).

## РЕЗУЛЬТАТЫ

Клинико-лабораторные (биохимические и генетические) данные пациенток, получающих МГТ по поводу ПНЯ, представлены в табл. 1.

С целью более наглядного сопоставления длин теломер у исследуемой группы (ПНЯ, n=33) в качестве референсных показателей мы привели аналогичные показатели здоровых женщин репродуктивного возраста (контрольная группа, n=24). Медиана возраста в данной группе составила 36 [ 30,0–40,0] лет, ИМТ=22 [ 20,0–27,0] кг/м², ФСГ=5,85 [ 4,75–8,55] мМЕд/л, длина теломер 10,8 [ 10,0–13,1] кБ.

Все пациентки с ПНЯ (n=24) с момента установления данного диагноза находились на ГЗТ, средний срок приема составил 5,5 [ 5,0–7,0] года.

Было установлена статистически достоверная умеренная обратная корреляционная связь (ρ=-0,434) между уровнем ФСГ и длиной теломер (p<0,001) (см. табл. 2), что математически подтверждает отрицательное влияние гипергонадотропного гипогонадизма на репликативный потенциал клетки. У пациенток с ПНЯ по сравнению с основной группой по данным биохимического анализа крови отмечается статистически значимая склонность к развитию нарушений углеводного обмена (в частности, предиабету) (р<0,043).

**Table:** Таблица 1. Данные пациенток с ПНЯ и без репродуктивной патологии Данные представлены в виде средних и стандартного отклонения (#) и/или медианы и интерквартильного размаха (##), а также относительных частот (%). * — различия показателей статистически значимы (p<0,05). Алгоритм расчета данных — см. раздел «Статистический анализ».

Параметр	Группа 1С ПНЯ и МГТ(n=33)	Группа 2Без ПНЯ и МГТ(n=24)	р
Возраст, лет#	33,7±7,2	34,2±2,7	< 0,001*
Возраст, лет##	35,5 [ 28,0–39,0]	36 [ 30,0–40,0]	< 0,001*
ИМТ, кг/м²#	23,5±4,3	25,2±6,0	0,5
ИМТ, кг/м²##	21,8 [ 19,9–27,8]	22 [ 20,0–27,0]	0,5
Предиабет, %	3,8	0,0	0,043*
Гипотиреоз, %	15,4	19,2	0,8
Альбумин, г/л#	44,4±2,5	44,7 ± 2,3	0,009*
Креатинин, мкмоль/л##	69,7 [ 63,7–75,9]	67,3 [ 63,6–72,2]	< 0,001*
Мочевина, ммоль/л##	6,3 [ 5,0–7,4]	5,5 [ 5,0–6,8]	0,08
Билирубин общий, ммоль/л##	10,0 [ 8,2–14,6]	9,5 [ 7,8–14,8]	0,2
Билирубин прямой, ммоль/л##	3,9 [ 3,4–5,6]	3,9 [ 2,6–5,2]	0,8
Глюкоза натощак, ммоль/л#	4,9±0,7	4,8 ± 0,6	0,6
ТГ, ммоль/л##	0,78 [ 0,59–0,98]	0,82 [ 0,64–1,00]	0,003*
ХС ЛПВП, ммоль/л##	1,8 [ 1,6–2,1]	1,8 [ 1,6–2,5]	0,006*
ОХС, ммоль/л##	4,58 [ 4,16–5,02]	4,86 [ 4,55–5,25]	0,3
ХС ЛПНП, ммоль/л##	2,6 [ 2,1–3,0]	2,7 [ 2,1–3,0]	0,1
Кальций общий, ммоль/л##	2,31 [ 2,27–2,36]	2,30 [ 2,27–2,36]	0,7
Кальций ионизированный, ммоль/л##	1,08 [ 1,05–1,11]	1,08 [ 1,05–1,12]	0,7
Фосфор, ммоль/л##	1,17 [ 0,97–1,29]	1,17 [ 1,10–1,31]	< 0,001*
АЛТ, ЕД/л##	12,00 [ 10,00–15,00]	14,00 [ 10,00–18,00]	< 0,001*
АСТ, ЕД/л##	16,00 [ 14,00–19,00]	17,00 [ 15,75–19,00]	< 0,001*
ГГТ, Ед/л##	16,0 [ 14,00–20,00]	16,00 [ 13,75–20,00]	< 0,001*
25(ОН)вит.D, нг/мл##	30,80 [ 23,30–41,90]	27,55 [ 22,18–35,50]	< 0,001*
Натрий, ммоль/л#	138,1 ± 3,0	138,1 ± 2,7	0,9
Калий, ммоль/л##	4,6 [ 4,0–5,0]	4,4 [ 4,1–5,1]	0,007*
Хлориды, ммоль/л#	105,0 [ 102,0–106,0]	105,0 [ 103,0–106,0]	0,1
ФСГ, мМЕд/л##	92,00 [ 91,00–95,00]	5,85 [ 4,75–8,55]	< 0,001*
ТТГ, мЕд/л##	2,1 [ 1,4–3,6]	1,9 [ 1,4–2,9]	0,9
HbA1c, %#	5,4 ± 0,4	5,4 ± 0,4	0,4
Длина теломер (кБ) ##	10,0 [ 7,9–10,7]	10,8 [ 10,0–13,1]	< 0,001*

**Table:** Таблица 2. Результаты корреляционного анализа взаимосвязи ФСГ и длины теломер

Показатель	Характеристика корреляционной связи
ρ	Теснота связи по шкале Чеддока	p
ФСГ — длина теломер	-0,434	Умеренная	< 0,001

## ОБСУЖДЕНИЕ

Несмотря на то что показательного улучшения репликативных (длина теломер) и биохимических маркеров у пациентов с ПНЯ на фоне заместительной терапии эстрадиолом не наблюдалось, обращает на себя внимание арифметически малая разница (вплоть до равенства) между статистически достоверными показателями как длины теломер (см. рисунок 1), так и биохимическими показателями (креатинин, фосфор, АЛТ, АСТ, ГГТ, калий) между основной и контрольной группами (см. табл. 1). Данный артефакт объясняется малым объемом выборки и ее неоднородностью (n=33+24=57). Не исключено, что в будущем для определения влияния половых стероидов на длину теломер необходим подбор пациенток с более длительной экспозицией эстрадиола, например, от 7 лет и более.

**Figure:**
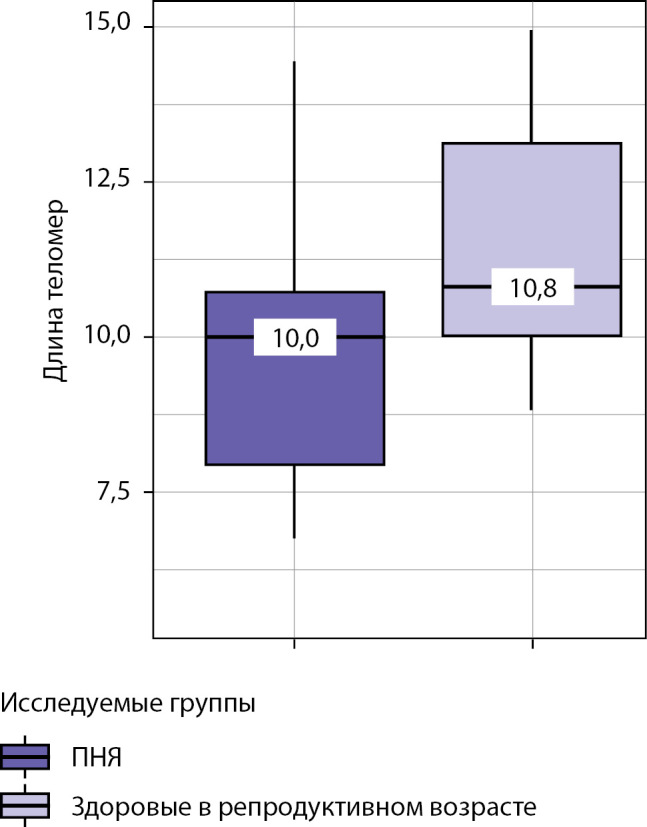
Рисунок 1. Анализ длины теломер в исследуемых группах.

Репрезентативность выборок

В оригинальном исследовании задействован относительно малый и неоднородный объем выборки контрольной и основной групп пациенток, что объясняется относительно редкой встречаемостью гипергонадотропного гипогонадизма аутоиммунного характера (ПНЯ) в общей популяции. Кроме того, на итоговый объем выборки серьезным образом повлияли финансово-техническая ограниченность исследования, принцип строгого соблюдения критериев включения и исключения (по данным анамнеза и катамнеза) во избежание искажения конечных результатов.

Сопоставление с другими публикациями

Так как ПНЯ характеризуется угрожающей тенденцией к особо стремительному развитию эстрогенного дефицита и фенотипа эстроген-дефицитной коморбидности, существует обоснованное мнение о необходимости назначения более высоких дозы эстрогенов (в отличие от пациенток в физиологической постменопаузе) для молодых больных, с поправкой на плохую переносимость (масталгия или мигрени) и патологические Эхо-признаки состояния эндометрия [2]; в этом случае должна быть произведена коррекция дозировки препарата в соответствии с индивидуальными потребностями и рисками. По мнению таких авторов, как Panay N. и соавт. [14], предпочтителен прием эстрадиола гемигидрата или эстрадиола валерата, так как они имеют более благоприятный защитный эффект на костную систему по сравнению с этинилэстрадиолом, входящим в состав комбинированных препаратов [14][15]. Для профилактики гиперплазии эндометрия у женщин, не подвергшихся гистерэктомии, эффективно использование микронизированного прогестерона или дидрогестерона [15]. Таким образом, не исключены усовершенствование и поиск других режимов заместительной терапии половыми стероидами для женщин с ПНЯ, обладающими как геро- (длина теломер), так и органопротекторными свойствами.

Клиническая значимость результатов

До последнего времени ПНЯ считалась необратимым состоянием, при котором шансы женщины как на осуществление репродуктивной функции, так и на здоровое долголетие считались практически равными нулю. Благодаря мультидисциплинарным изысканиям акушеров-гинекологов, эндокринологов в тактику ведения таких пациентов удалось внедрить заместительную терапию половыми стероидами — не только симптоматически, но и патогенетически обоснованный метод лечения. Поскольку ПНЯ сочетается с другими серьезными эндокринопатиями в составе аутоиммунного полигландулярного синдрома (1, 2 и 4 типов), то определение длины теломер само по себе является многообещающим методом скрининга, с целью определения персонализированной тактики диагностики и лечения.

Ограничения исследования

Главными факторами, ограничивающими масштаб и внешнюю валидность данного исследования, являлись относительная узость и неоднородность выборки, что объясняется высокой финансово-технической емкостью технологий определения длины теломер, доступных на настоящий момент специалистам в Российской Федерации.

Направления дальнейших исследований

В качестве показательного, статистически значимого пути получения биоматериала мы предполагаем в будущем проведение слепых плацебо-контролируемых рандомизированных клинических исследований с проведением биопсии (пункции) яичников для последующего определения длины теломер до и после многолетнего приема МГТ. Осуществление такого рода дизайна на практике пока остается затруднительным с этической и финансово-технической точки зрения.

## ЗАКЛЮЧЕНИЕ

Результаты данного исследования являются показательным, но в то же время сами по себе не исчерпывающи. Окончательное решение вопроса о влиянии заместительной терапии половыми стероидами на длину теломер у женщин в рамках концепции «здорового долголетия» будет достигнуто при условии проведения многолетних двухмоментных слепых плацебо-контролируемых рандомизированных клинических исследований с применением различных модификаций и дозировок эстрадиола.

## ДОПОЛНИТЕЛЬНАЯ ИНФОРМАЦИЯ

Источник финансирования. Исследование проводится в рамках Государственного задания: «Влияние эпигенетических факторов на течение менопаузы у женщин с эндокринопатиями аутоиммунного генеза в рамках формирования модели “здорового старения”», регистрационный номер АААА-121030100033-4.

Конфликт интересов. Авторы декларируют отсутствие явных и потенциальных конфликтов интересов, связанных с публикацией настоящей статьи.

Участие авторов. Все авторы одобрили финальную версию статьи перед публикацией, выразили согласие нести ответственность за все аспекты работы, подразумевающую надлежащее изучение и решение вопросов, связанных с точностью или добросовестностью любой части работы.
